# Melodious tuning of microbial dynamics in biofloc, cage, and pond culturing system: a study on *Pangasius pangasius* fish gut microbiome

**DOI:** 10.3389/fmicb.2024.1434312

**Published:** 2024-07-10

**Authors:** Foram D. Vala, Smit R. Lende, Vishal K. Solanki, Kiran Jora, Riya Desai, Parth Sharma, Neelam Nathani, Chandrashekar Mootapally

**Affiliations:** ^1^Department of Aquaculture, College of Fisheries Science, Kamdhenu University, Veraval, Gujarat, India; ^2^Centre of Excellence in Aquaculture, Kamdhenu University, Ukai, Gujarat, India; ^3^School of Applied Sciences and Technology (SAST-GTU), Gujarat Technological University, Chandkheda, Ahmedabad, Gujarat, India

**Keywords:** gut microbiome, aquaculture systems, Pangasius, community differences, hostmicrobiome

## Abstract

Aquaculture, a rapidly expanding sector, meets the global surging demand for aquatic food. Pangasius, a highly valued freshwater species, has seen a significant increase in demand due to its adaptability and potential for high yields, making it a promising candidate for aquaculture in India. This study investigates the gut microbiome composition of *Pangasius pangasius* fish cultured in three different systems (biofloc, cage, and pond). Metagenomic DNA extraction and 16S rRNA gene-targeted sequencing were performed. Outcomes revealed distinct microbial compositions across culture types, with significant differences in species richness and diversity, specifically in the biofloc system, compared to cages and ponds. Taxonomic analysis identified prevalent phyla such as Firmicutes and Fusobacteriota, with varying abundances among culture systems. The genus-level analysis highlighted dominant genera such as *Cetobacterium* and WWE3. Functional profiling indicated differences in enzymatic activity and metabolic pathways, emphasizing each culture sample type's unique microbial community structures. Notably, the microbiota from BF samples exhibited significant differences and unique metabolic pathways compared to the microbiota from C and P samples, which showed greater similarity and shared several common metabolic pathways. These findings highlight substantial differences in microbial diversity across the culturing systems, reflecting the microbiota's ability to adapt to specific environments and their potential role in promoting fish growth within those environments. Overall, this study provides insights into the gut microbiome diversity and functionality in *Pangasius pangasius* across different aquaculture environments, contributing to a better understanding of host–microbe interactions and aquaculture management strategies.

## Graphical Abstract

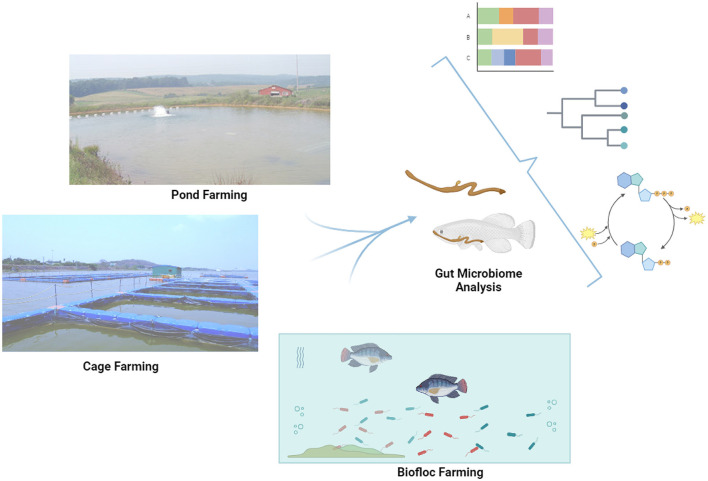

## Introduction

Aquaculture is a rapidly growing and important producing sector with the potential to reach the exploding demand for aquatic food. Global aquaculture production reached 122.6 million tons, including 87.5 million tons of aquatic animals, with a market value of ~$ 264.8 billion. Since the mid-1980's, India topped the inland fisheries for the first time with a production of 1.8 million tons; on the other hand, global consumption of aquatic foods increased at an average annual rate of 3.0% from 1961 to 2019 (Fisheries, 2022). Disease management is the main topic of concern in the aquaculture sector, as this is the rapidly growing food-protein-producing sector all over the world (Bacher, 2015). Growing concern over the antibiotic compounds found in foods and an increase in antibiotic-resistant microbes have led to an interest in alternatives to antibiotics, such as probiotics, for the treatment and prevention of disease (Pereira et al., 2022). Probiotics are live microbial feed supplements that benefit the host by improving its intestinal balance. Numerous microorganisms live in the alimentary tract, playing an important role in fish nutrition, health, and physiological processes (Nayak, 2010; Luan et al., 2023).

Pangasius, which can fetch rupees 150–200 per kg in the local/domestic market, is being reinforced and promoted as a result of the rising demand for freshwater species in domestic markets. The species can gain weight up to 1–1.5 kg in 1 year, with annual yields expected at ~10 to 15 tons per hectare. Pangasius production was estimated to be ~400,000 to 425,000 metric tons in 2016. The inland production of catfish (Wallago Attu. and Pangasius) in India reached 0.61 lakh tons in 2017 (Fisheries, Ministry of Fisheries, and Government of India 2020). Pangasius was introduced in India in 1995 (Mugaonkar et al., 2019), and various studies have revealed that the development of different value-added products from Pangasius has high global acceptance and commercial potential. Pangasius is a very hardy species and can withstand long-range temperatures, salinity, low oxygen, and high turbidity (Gupta, 2016). In India, Pangasius has been described as an appropriate candidate species for both monoculture and polyculture with carp, either in ponds or in net cages. By 2030, freshwater species such as pangas catfish and carp are projected to constitute the majority (62%) of world aquaculture production (Fisheries, 2022).

Given the rising demand for Pangasius fish in aquaculture methods, a proper understanding of the physiology and dietary needs of the fish will depend greatly on the description of the typical gut flora of the fish. The alimentary tract is a complex ecosystem containing a huge number of inhabitant microorganisms. The abundance of gut bacteria in fish significantly influences their physiology and health (Yukgehnaish et al., 2020). The link between gut microbiota and the adjoining environment is reflected in the functional potential of the bacterial community (Abubucker et al., 2012). As a result, the purpose of this study is to investigate the bacterial communities present in the gut of *Pangasius pangasius* taken from biofloc (BF), cage (C), and pond (P) systems. The main objective that can be accomplished through metagenomics includes a study of environmental microbes, bioprospecting (finding new genes with the desired bioactivity), and related function with the phylogeny of a given sample as well as the developmental profile of the community structure and function (Thomas et al., 2012).

Considering the increased demand for Pangasius fish in aquaculture practices, it is essential to study aquatic bacterial flora, which plays an important role in water productivity. Some active species are responsible for fish disease, so it is essential to know the presence of microflora in the water body as well as in the fish intestine in that setting. The microbiota found in the gut is a reflection of microorganisms from the surrounding environment or the food consumed that can survive and reproduce within the intestinal tract. Furthermore, the description of the typical intestinal flora of the fish will be of great importance in the proper understanding of the physiology and nutritional requirements of the fish. Therefore, the goal of this study is to investigate the presence of distinct bacterial diversity in the gut of *Pangasius pangasius* collected from different culturing systems, illustrating their capacity to provide distinct advantages within their respective environmental contexts.

## Materials and methods

### Ethical statement

The research conducted in this study involved fish specimens and ethical approval was obtained from the research committee (Letter No. KU/DR/U-2/OFFICE ORDER/1089/2020 dated 06.10.2022) of Kamdhenu University.

### Experimental design and sample collection

Fish (*Pangasius pangasius*) samples were collected from two different places in Gujarat from three different culture systems [viz. BF, C, and P]. C and P fish samples were sampled from Selud village (21.18° N, 73.64° E) situated in Tapi district on 19 January 2022, while BF fish samples were collected from Idar village (23.83° N, 73.00° E) in Sabar Kantha district on 3 March 2022. To analyze the gut microbiome, samples from each culture system were randomly collected in triplicate. Three specimens of healthy adult fishes were collected from each culture system, with weights ranging from 1,300 to 1,500 g for BF (F4, F5, F6), 600 to 800 g for C (F13, F14, F15), and 700 to 900 g for P (F16, F17, F18), and transferred to the sample collection box. Dissection was carried out aseptically in laminar air flow using sterile surgical tools in the laboratory. Each fish gut was dissected, and hindgut fluid was collected and stored at −80°C to be analyzed later.

### DNA isolation and quantification

Metagenomics DNA was extracted using QIAamp^®^ DNA Stool Mini Kit (QIAGEN) according to the manufacturer's protocol. The extracted DNA was quantified using the QIAxpert instrument, followed by Qubit^®^ dsDNA BR Assay Kit (Thermo Fisher Scientific, USA), and quality was assessed through electrophoresis on 1% agarose gel. The metagenomics DNA was examined with the help of PCR by using the universal primer for the 16S rRNA gene (27F 5'-AGAGTTTGATCCTGGCTCAG-3', 1492R 5'-GGTTACCTTGTTACGACTT-3'; Miller et al., 2013).

### V3–V4 amplification and sequencing

The amplicon library was prepared as per the standard protocol of 16S metagenomics library preparation for the Illumina MiSeq platform, briefly described as follows (Illumina, USA). PCR amplification was carried out to target the V3 and V4 regions of the 16s rRNA gene using 341F (5'-CCTACGGGNGGCWGCAG-3') and 805R (5'-GACTACHVGGGTATCTAATCC-3') primers. Libraries were verified on Bioanalyzer (Agilent, USA) and quantified using Qubit^®^ dsDNA HS Assay Kit (Thermo Fisher Scientific, USA). Sequencing was carried out on the Illumina MiSeq platform using MiSeq reagent cartridge reagent kit (500-cycle) 2 × 250 v2 chemistry pair-end sequencing, as per the Illumina MiSeq system guide.

### Data analysis

Sequence read quality was assessed using FastQC (http://www.bioinformatics.babraham.ac.uk/projects/fastqc). Based on the observed quality, the reads were filtered out with the following parameters: All forward/reverse reads were trimmed by seven bases from the left; from the right end, the reads were truncated at a length of 240 for forward and 200 for reverse. The reads were further pre-processed with DADA2 for denoising and eliminating chimera sequences and duplicates. Based on the observed features, a sampling depth of 99 was maintained, which resulted in the omission of two samples (F4 and F15 of BF and C, respectively), which had lower features compared to the selected depth, from the microbial diversity estimation and correlation. The predicted ASVs were normalized by the minimum number of feature sequences in a sample from each of the study groups, respectively. Read pre-processing and taxonomic classification were performed in the QIIME2 v2022.8 framework (Bolyen et al., 2019) using the pre-trained SILVA classifier for the V3–V4 region of the 16S rRNA gene. Beta diversity was calculated using weighted and unweighted UniFrac metrics to compute the distribution of the samples of studied groups.

The microbial taxonomy was studied for statistical differences (*p* < 0.05) between the studied groups, and the core microbiota was compared using Microbiome Analyst. Functional attributes corresponding to the observed microbiota were assessed using the q2-PICRUST 2:2021.11 plugin (Douglas et al., 2020). The statistical differences between the samples were studied by performing the Kruskal–Wallis test in STAMP v2.1 (Parks et al., 2014).

## Results

### Taxonomic content of microbial community

A total of 6,928,665 reads were obtained with an average length of 328.92. All the observed ASVs were classified as bacteria, archaea, and eukaryotes. Bacteria were distributed in 16 phyla and 51 genera. The rarefaction analysis revealed a higher number of observed species in BF as compared to the C and P (Supplementary Figure 1). The vertical axis shows the Shannon diversity, while the horizontal axis shows the sequencing depth considered for diversity calculation.

### Correlation of microbial community between BF, C, and P environments

**Figure 1:**
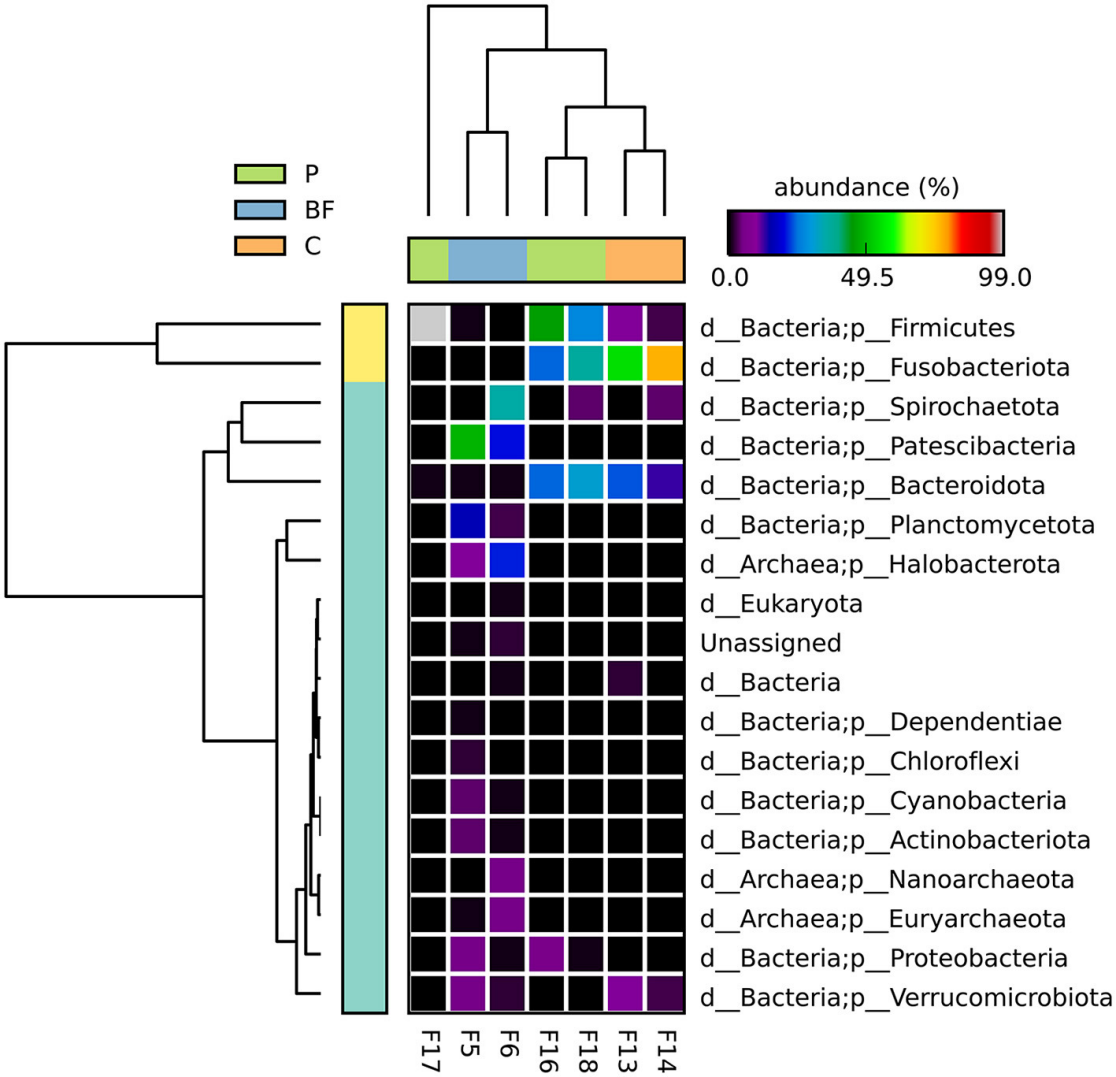
Bacterial abundance (in %) of all the observed phyla among the studied samples, depicting the dominant phylum and variability in phyla abundances across culturing system type. There was no significant difference (*p* < 0.05) at the phyla level among the studied groups.

The microbial community was assessed at the phylum level to see if there was any impact of the type of culture system on the fish gut microflora. There was a clear difference across the three systems, with Firmicutes and Bacteroidota phyla more dominant in the C and P systems, while in the BF culture in the bacteria Patescibacteria, while for the samples of BF culture, Patescibacteria and Halobacteriota were among the dominant phyla, followed by Planctomycetota and Spirochaetota in high abundance; however, these were not consistently identified as abundant phyla across the tested samples from the same group (Figure 1). Looking at the statistical significance in variations of phyla between the three studied groups, it was observed that the Patescibacteria, Fusobacteriota among bacteria, and Halobacterota among archaea were significantly varying. Notably, no common phyla were consistently found across all sampled environments. The observed phyla-level microbial abundance provided insights into the diverse microbial compositions within the fish gut across different culture systems.

**Figure 2:**
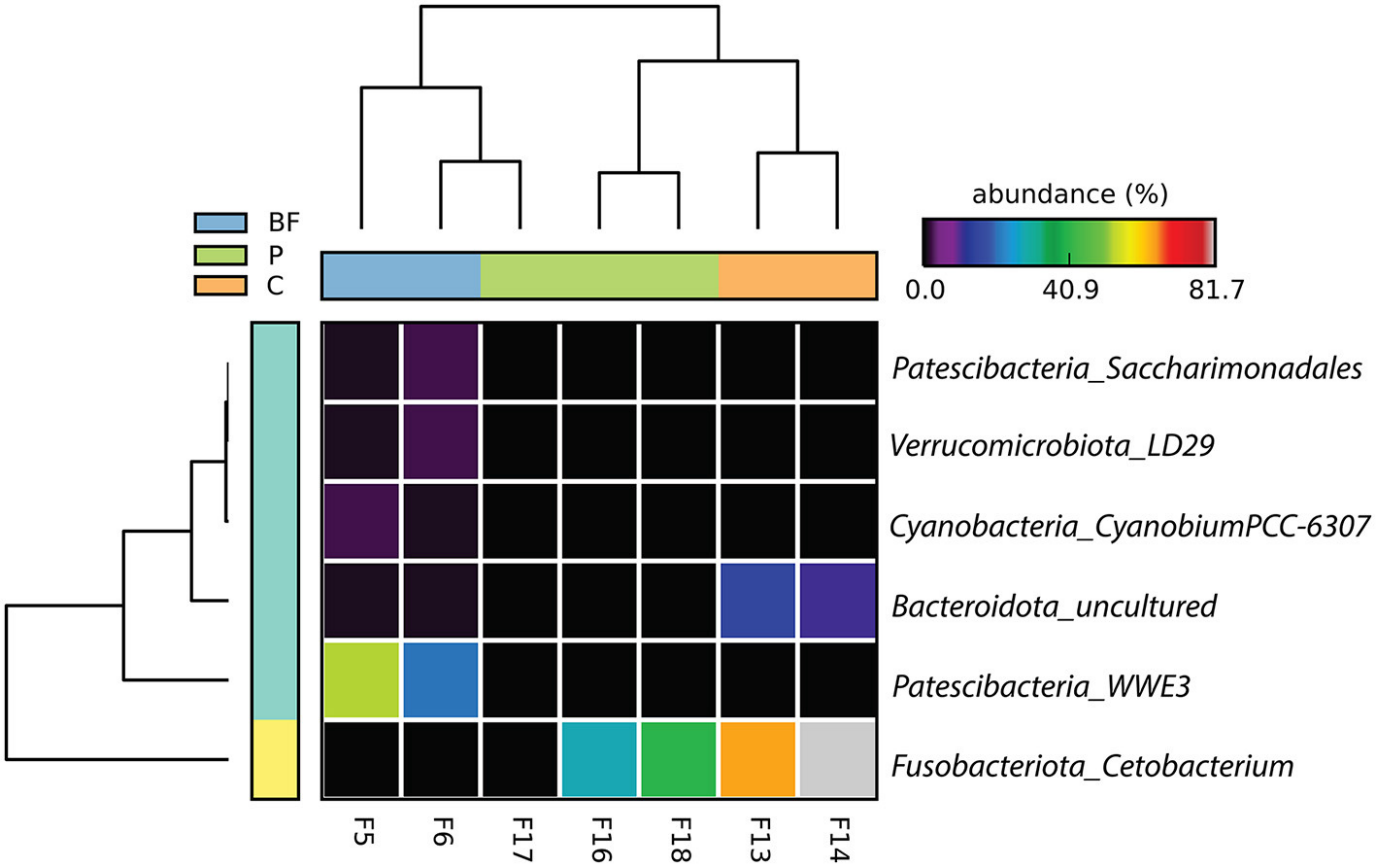
Genus-level bacterial proportion significantly differs (*p* < 0.05) across the three studied culturing systems. The label on the right corresponds to phylum_genera.

At the genus level, *saccharimonadales* and *WWE3* from the Patescibacteria phyla, *LD29* from Verrucomicrobiota*, Cetobacterium* from the Fusobacteriota*, Cyanobium* from Cyanobacteria, and some uncultured Bacteroidota were among the dominant and significantly different genera among the samples, as depicted in Figure 2. Specifically, *Cetobacterium* exhibited high dominance in both C- and P-cultured fish samples, while *WWE3* was uniquely prevalent in BF samples. These findings at the genus level underscore the distinct microbial composition variations present among the different culture systems, emphasizing the prominence of specific genera in the C and P culture systems more similar compared to the BF environment.

**Figure 3:**
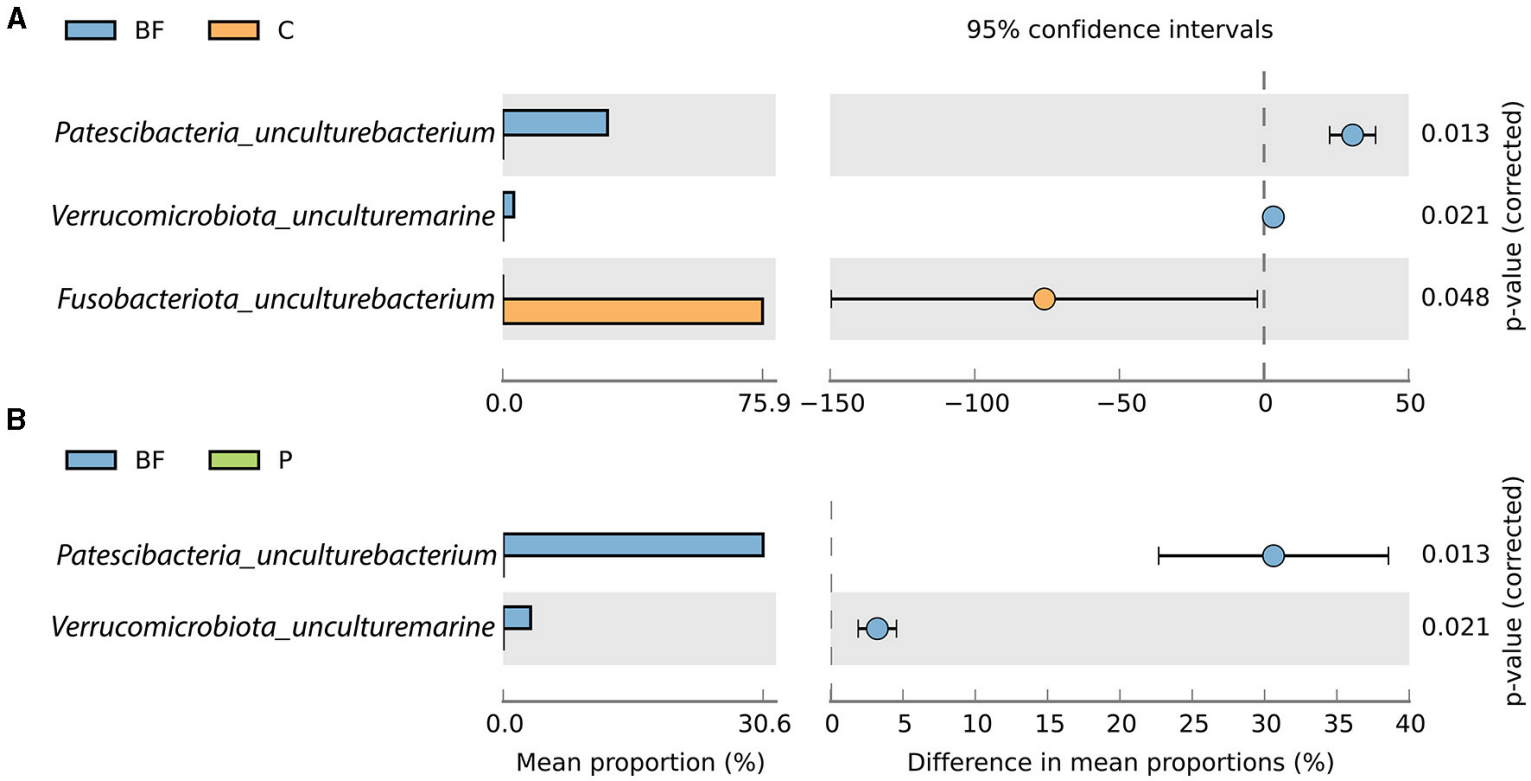
Proportion (in %) of significantly (*p* < 0.05) varying species between **(A)** BF and C, and **(B)** BF and P.

Furthermore, genus-level classification showed that uncultured bacterium representation from Patescibacteria and *Verrucomicrobiota* were significantly prevalent in BF-cultured fish gut samples species and were absent in the P- and C-cultured fish gut samples as depicted in Figure 3. In contrast, *Fusobacteriota_uncultured bacterium* was notably predominant in C-cultured samples and absent in the BF-cultured samples (Figure 3A).

**Figure 4:**
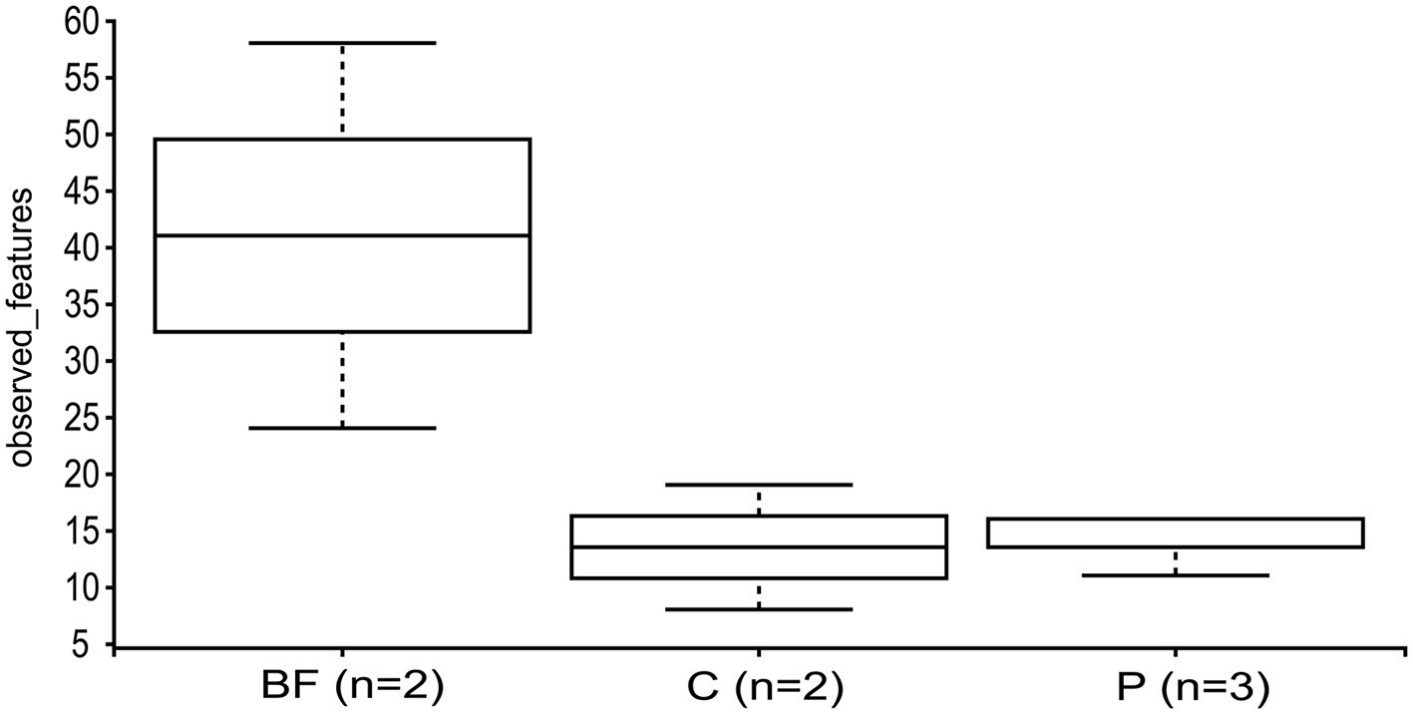
Alpha-diversity, depicted by the observed features and assessed via the Kruskal–Wallis test across the studied samples, considering the sample type as a variable.

The alpha diversity measured using the Kruskal–Wallis test revealed that the BF-cultured samples had a higher species richness than the C and P samples (Figure 4). Considering fish weight as a variable, alpha-diversity analysis showed a relatively higher Shannon entropy in the higher weight group (1,300–1,500) compared to the other two weight groups; however, the difference was not statistically significant (Supplementary Figure 2).

### Functional attributes based on enzyme and pathway prediction

The functional profiling of the bacteria present in all gut samples was explored based on the 16S rRNA gene profiling using picrust2: 2021.11. It correlates the taxonomic classification of bacteria to their metabolic role in the host.

**Figure 5:**
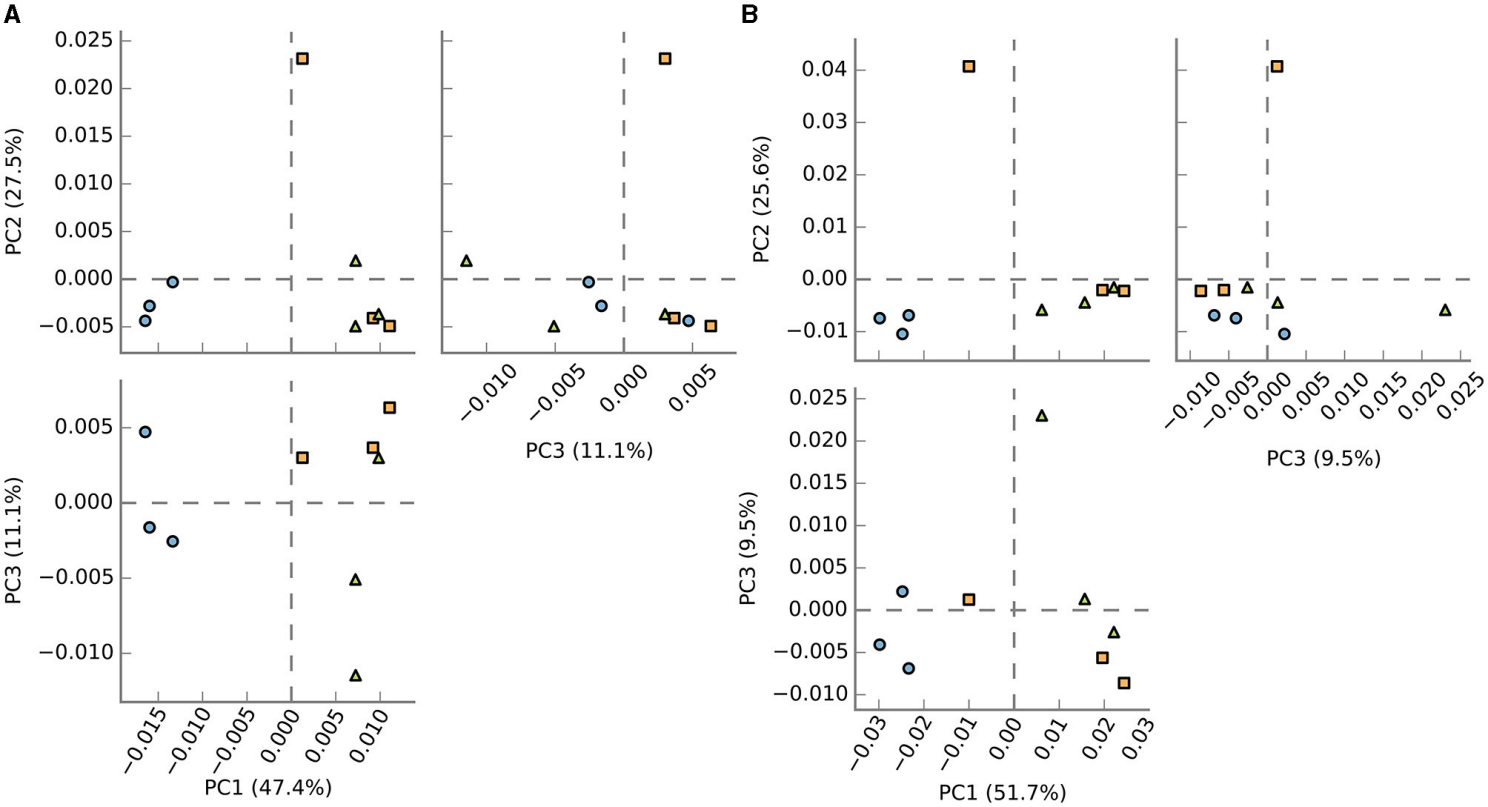
PCoA analysis of samples based on the **(A)** enzyme activity and **(B)** pathways. It demonstrated that BF (blue-filled circles) samples clustered more tightly and separately compared to C (orange-filled squares) and P (green-filled triangles), which, though clustered near to each other, were overall more scattered.

A comparative analysis of the relative similarities and differences among the samples was conducted, and the outcomes are depicted in Figure 5A. The results revealed significant disparities in the enriched communities of BF, C, and P samples, as depicted in the upper and lower principal component analysis (PCA) plots. Interestingly, the PCA plots demonstrated a closer relationship between the C and P fish gut samples, indicating a higher degree of similarity between these two environments, which was in line with the observed microbial composition. In contrast, the BF-cultured fish gut samples appeared notably distinct when compared to the C and P samples, signifying a substantial dissimilarity in their microbial compositions and highlighting the unique nature of the BF culture system in terms of its microbial community structure.

The pathway prediction was carried out, which showed the existence of different pathways in the samples. The PCA plot results (Figure 5B) show that based on the pathways as well, the BF samples clustered differently from the other two groups. Overall, the C and P samples show more similarity among the studied samples.

**Figure 6:**
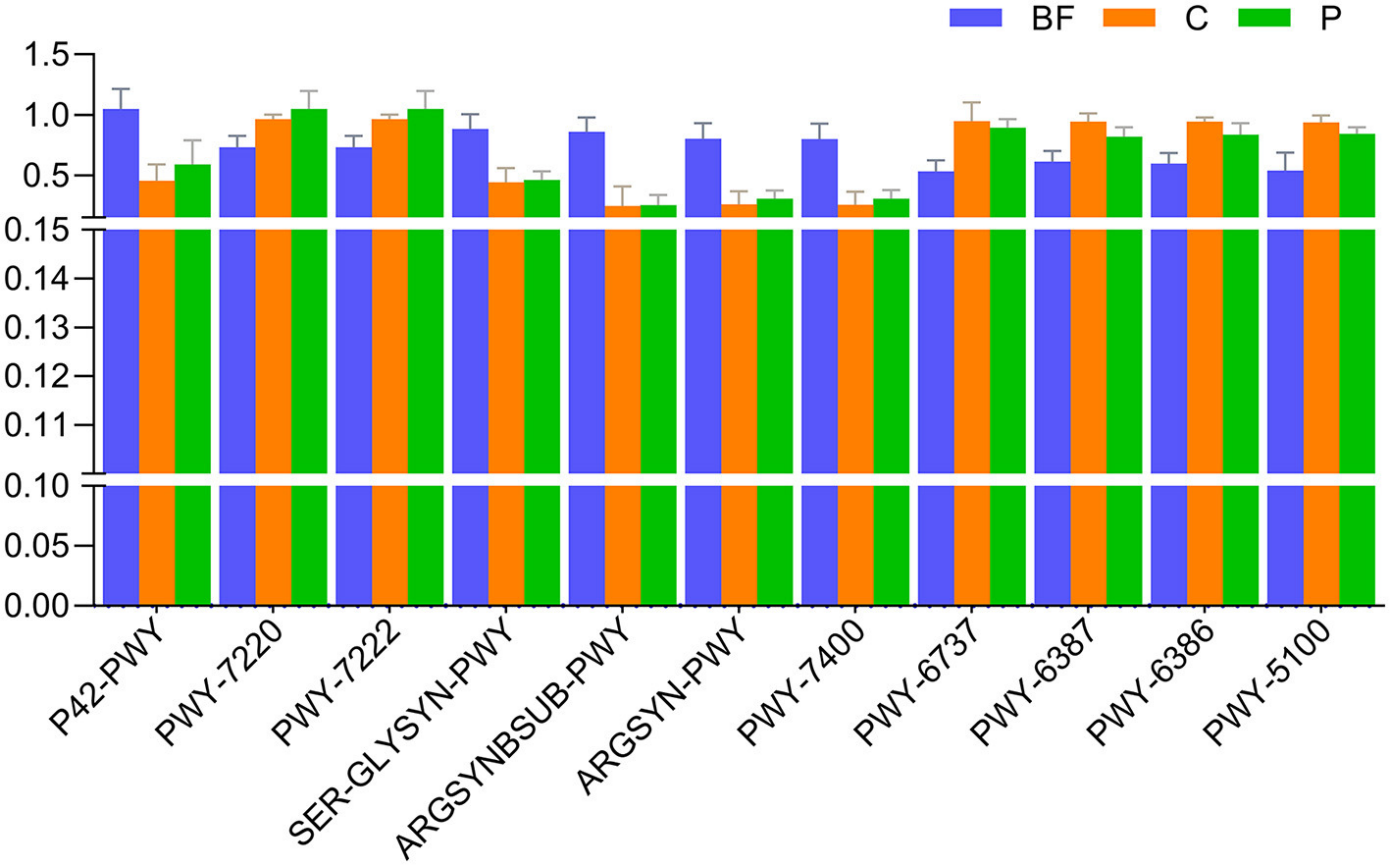
Comparison of top pathways (with mean relative frequency >1%) significantly varying (*p* < 0.05) across the studied culturing environments.

Specific metabolic pathways such as incomplete reductive TCA cycle (P42-PWY), adenosine deoxyribonucleotides *de novo* biosynthesis II (PWY-7220), guanosine deoxyribonucleotides *de novo* biosynthesis II (PWY-7222), the super pathway of L-serine and Glycine biosynthesis (SER-GLYSYN-PWY), L-arginine biosynthesis (ARGSYNBSUB-PWY, ARGSYN-PWY), dTDP-beta-L-4-epi-vancosamine biosynthesis (PWY-7400), starch degradation (PWY-6737), UDP-N-acetylmuramoyl-pentapeptide biosynthesis I (meso-diaminopimelate containing; PWY-6387), UDP-N-acetylmuramoyl-pentapeptide biosynthesis II (lysine-containing; PWY-6386), and pyruvate fermentation to acetate and lactate II (PWY-5100) exhibited significant differences across all the samples (Figure 6).

Furthermore, while examining unique pathways in each of the studied culture systems, there was one pathway each unique to the BF and P system pertaining to degradation and salvage activities. In contrast, the C system had ~7 unique pathways, most of which were biosynthesis-related pathways (Table 1). As we had observed the C and P systems to be closer in the microbial content, we also had a manual look into the common pathways in these two systems, which revealed 10 pathways commonly present in these two systems in comparison to the BF system (Table 2). These metabolic pathways represent functional aspects of microbial communities and their activity within the fish gut samples. The identification of these distinct metabolic pathways emphasizes the variability and unique metabolic capabilities present among the samples, contributing to a deeper understanding of the functional differences across the microbial communities in each sample.

**Table 1:** Unique pathways observed across the studied culturing environments.

**Culturing environment**	**Pathways code**	**Pathways name**
Biofloc	PWY-6572	Chondroitin sulfate degradation I (bacterial)
Cage	PWY0-1241	ADP-L-glycero-β-D-manno-heptose biosynthesis
	PWY0-1415	Superpathway of heme biosynthesis from uroporphyrinogen-III
	PWY-5920	Superpathway of b heme biosynthesis from glycine
	PWY-7371	1,4-dihydroxy-6-naphthoate biosynthesis II
	PWY-6263	Superpathway of menaquinol-8 biosynthesis II
	P124-PWY	Bifidobacterium shunt
	PWY0-1479	tRNA processing
Pond	PWY-7094	Fatty acid salvage

**Table 2:** Common pathways between the cage and pond-cultured fish gut samples.

**Code**	**Pathway**
P221-PWY	Octane oxidation
PWY-5747	2-methylcitrate cycle II
PWY0-42	2-methylcitrate cycle I
PWY-7347	Sucrose biosynthesis III
CHLOROPHYLL-SYN	3,8-divinyl-chlorophyllide a biosynthesis I (aerobic)
PWY-5531	3,8-divinyl-chlorophyllide a biosynthesis II (anaerobic)
PWY-7159	3,8-divinyl-chlorophyllide a biosynthesis I (aerobic, light-dependent)
SUCSYN-PWY	Sucrose biosynthesis II
PWY-181	Photorespiration I
PWY-6478	GDP-D-glycero-α-D-manno-heptose biosynthesis

## Discussion

The purpose of this investigation was to look at the variety of microbes found in fish gut samples raised in three distinct environments. Our observations of multiple ASVs that were distinguishable down to the species level provided important insights for our investigation. Certain ASVs demonstrated distinct and/or elevated abundances in the microbial populations across various culture group systems that were the subject of the investigation. Understanding the physiology and function of microbial communities in the fish gut across various culture systems depends on this degree of specificity in identifying particular microbial variations at the species level. Research using next-generation sequencing (NGS) methods has revealed the presence of multiple well-known bacterial phyla in fish gastrointestinal tracts. Proteobacteria, Firmicutes, Actinobacteria, Fusobacteria, Bacteroidetes, and Verrucomicrobia have all been documented in earlier investigations among the microbial taxa that predominate in the fish gut (Desai et al., 2012; Carda-Diéguez et al., 2014; Li et al., 2015; Talwar et al., 2018). The characterization of bacteria has revealed information about the many microbial communities that thrive in the fish gut, as well as their microbial diversity and their relative abundance.

As we have witnessed, fish guts contain a comparatively large concentration of fusobacteria (Van Kessel et al., 2011; Di Maiuta et al., 2013). Fusobacteria are Gram-negative rod-shaped bacteria that produce butyrate (Larsen et al., 2014). The fermentation of dietary fiber results in the production of butyric acid by the intestinal microbiome of fish on the epithelium. In addition to having immunomodulatory and anti-inflammatory properties, it serves as the primary medium for gut bacteria to breathe (Rimoldi et al., 2016; Terova et al., 2016). Additionally, Fusobacteria can generate substantial amounts of vitamin B12 (Roeselers et al., 2011; Larsen et al., 2014; Udayangani et al., 2017) which can have a significant effect on the fish gut. One study indicates that Fusobacteria, which produce short-chain fatty acids (SCFAs), play a crucial role in enhancing the epithelial barrier and modulating the inflammatory response (Zhang et al., 2020). Furthermore, research has demonstrated that dietary SCFAs can mitigate oxidative stress and augment mucosal immune responses in crucian carp (Li et al., 2022). Bacteroides have the capability of assimilating polysaccharides and metabolizing carbohydrates, and they play a role in breaking down complicated compounds into simpler ones in the host gut. It has been observed that Bacteroides produce vitamin B1 in freshwater fish such as carp, goldfish, and tilapia (Xia et al., 2014; Udayangani et al., 2017). The Bacteroides phylum's contributors are renowned for being involved in the fermentation of carbohydrates, which results in the production of a group of volatile fatty acids that are reabsorbed via the digestive tract and used as an energy source (Aisyah et al., 2019). One of the studies shows that resveratrol boosts the beneficial gut bacteria Bacteroides, enhancing gut health. Transferring this improved microbiota to obese mice helps them regulate blood sugar levels more effectively (Zhang et al., 2022). The anaerobic microorganisms that flourish in digestive tracts have demonstrated that fish gut contains Planctomycetota. Those microbes have an impact on complicated chemical metabolism. Planctomycetota serves as decomposers of sulfated polysaccharides produced by kelp (Bengtsson and Øvreås, 2010; Orellana et al., 2022). Planctomycetota have also been reported to play a role in the core microbiome that might be conserved within *C. virginica* and other shellfish (King et al., 2012). One study noted that an increase in Verrucomicrobia, particularly Luteolibacter, benefits fish gut health by enhancing immune responses, regulating microbial communities, and promoting mucosal repair (Wu et al., 2022). The Firmicutes phylum is broadly distributed across natural environments and primarily consists of Gram-positive bacteria capable of forming spores. These bacteria are essential components of diverse ecosystems, especially in microbial communities responsible for breaking down plant cellulose and decomposing carbohydrate polymers (Liu et al., 2019). It has been noted that Bacillus was crucial to the fish intestine's ability to produce cellulase. It has also been noted that Bacillus are crucial to the GI tract's ability to metabolize carbohydrates in grass carp (Deb et al., 2020). One study suggests that Proteobacteria and Firmicutes contribute to digestion in fish species such as parrotfish, snapper, and surgeonfish by producing a diverse array of enzymes (Smriga et al., 2010; Dulski et al., 2018).

This investigation used a comparative prevalence analysis of microbial metabolic pathways across various samples to elucidate their functional roles. Distinct metabolic profiles were identified, with a noteworthy observation of the BF-associated pathway PWY-6572, implicated in bacterial degradation of chondroitin sulfate I. Notably, gut samples obtained from C-cultured fish displayed a predominance of unique pathways, including PWY0-1241 (biosynthesis of ADP-L-glycero-β-D-manno-heptose), PWY0-1415 (super pathway for heme biosynthesis from uroporphyrinogen-III), PWY-5920 (super pathway for heme biosynthesis from glycine), PWY-7371 (biosynthesis of 1,4-dihydroxy-6-naphthoate II), PWY-6263 (super pathway II for menaquinol-8 biosynthesis), P124-PWY (Bifidobacterium shunt), and PWY0-1479 (tRNA processing). Conversely, gut samples from P-cultured fish exhibited a distinct enrichment of pathway PWY-7094, associated with fatty acid salvage. These observations underscore the intricate metabolic landscapes shaped by varying environmental conditions and culture techniques within the gut microbiomes of fish.

The dominant metabolic landscape was characterized by prevalent biosynthesis pathways, notably the super pathways for heme biosynthesis originating from both uroporphyrinogen-III and glycine, 1,4-dihydroxy-6-naphthoate biosynthesis II, and menaquinol-8 biosynthesis II. This observation underscores a pronounced emphasis on metabolite synthesis within the gut microbiome. Furthermore, the identification of pathways associated with tRNA processing offers valuable insights into the critical role of these molecular adapters. tRNAs function as essential intermediaries in protein synthesis, facilitating the translation of transcribed messenger RNA (mRNA) sequences derived from protein-coding genes (Kirchner and Ignatova, 2015). Chondroitin sulfates and dermatan sulfate, key elements of the connective tissue matrix in skin and cartilage, were involved in this pathway. These specific functional pathways show potential in enhancing the immune response of fish, offering possible protection against harmful effects (Habicher et al., 2015; Hou et al., 2023).

Shared functional pathways were identified in both C- and P-cultured fish gut samples, including P221-PWY (octane oxidation), PWY-5747 (2-methylcitrate cycle II), PWY0-42 (2-methylcitrate cycle I), PWY-7347 (sucrose biosynthesis III), CHLOROPHYLL-SYN (3,8-divinyl-chlorophyllide a biosynthesis II—anaerobic), PWY-5531 (3,8-divinyl-chlorophyllide a biosynthesis II—anaerobic), PWY-7159 (3,8-divinyl-chlorophyllide a biosynthesis I—aerobic, light-dependent), SUCSYN-PWY (sucrose biosynthesis II), PWY-7374 (1,4-dihydroxy-6-naphthoate biosynthesis I), PWY-181 (photorespiration I), and PWY-6478 (GDP-D-glycero-α-D-manno-heptose biosynthesis). These conserved pathways across different environmental conditions offer insights into fundamental metabolic processes and adaptive strategies in fish gut microbiomes under varying cultivation practices. The identification of shared pathways highlights potential key contributors to metabolic stability and environmental adaptability in the fish populations studied.

## Conclusion

In the present study, we analyzed the microbiota abundance in Pangasius fish from three different culture types and conducted comparative assessments. We observed distinct variations in alpha-diversity and beta-diversity profiles among the studied fish gut samples. Notably, the microbiota from BF samples exhibited significant differences and unique metabolic pathways compared to the microbiota from C and P samples, which showed greater similarity and shared several common metabolic pathways. These findings highlight the substantial differences in microbial diversity across the culturing systems, reflecting the microbiota's ability to adapt to specific environments and their potential role in promoting fish growth within those environments.

## Data availability statement

The raw metagenomics sequence of the BF, C, and P samples have been deposited to the NCBI SRA under the BioProject PRJNA1091202.

## Ethics statement

The animal study was approved by Ethical Research Committee-Kamdhenu University (Letter No. KU/DR/U-2/OFFICE ORDER/1089/2020 dated 06.10.2022). The study was conducted in accordance with the local legislation and institutional requirements.

## Author contributions

FV: Investigation, Methodology, Writing – original draft. SL: Conceptualization, Methodology, Writing – review & editing. VS: Investigation, Methodology, Writing – original draft. KJ: Investigation, Methodology, Writing – original draft. RD: Visualization, Writing – original draft. PS: Formal analysis, Writing – original draft. NN: Formal analysis, Writing – review & editing. CM: Visualization, Writing – review & editing.
